# Hepatic Epithelioid Hemangioendothelioma and the Danger of Misdiagnosis: Report of a Case

**DOI:** 10.1155/2013/243939

**Published:** 2013-02-28

**Authors:** Kyriakos Neofytou, Andreas Chrysochos, Nikolas Charalambous, Menelaos Dietis, Christos Petridis, Charalampos Andreou, Athanasios Petrou

**Affiliations:** ^1^Department of Surgery, Nicosia Government Hospital, Palaios Dromos Lefkosias-Lemesou, No. 215, 2029 Strovolos, Nicosia, Cyprus; ^2^Department of Radiology, Nicosia Government Hospital, Palaios Dromos Lefkosias-Lemesou, No. 215, 2029 Strovolos, Nicosia, Cyprus

## Abstract

Malignant hepatic epithelioid hemangioendothelioma (HEHE) is a rare malignant tumor of vascular origin. Nonspecific symptoms and the absence of experience of surgeons, radiologists, and histopathologists due to the rarity of HEHE make the diagnosis of this entity very challenging. Misdiagnosis is not a rare event, and the consequences of such an event are catastrophic. We report a case of a patient suffering from HEHE in which the initial diagnosis was hepatocellular carcinoma (HCC). The presence of normal laboratory values, liver function tests, tumor markers along with the absence of a chronic liver disease, or any other predisposing factors for HCC, was in contrast with the diagnosis of HCC. Clinical suspicion drove us to the repetition of a liver biopsy and the reevaluation of the sample by a more experience histopathology department in liver tumors. The last biopsy confirmed the diagnosis of HEHE, and the patient escaped any unnecessary treatment for a nonexisting HCC.

## 1. Case Presentation

A forty-nine-year old female patient presented with a right upper quadrant pain. Based on her medical history, the patient underwent thyroidectomy 6 months before because of a papillary thyroid cancer that was not invading the thyroid capsule (TNM staging PT1, PNX, PMX Anatomic stage/Prognostic groups 45 years, and older Stage 1).

All of the patient laboratory tests were within normal limits including alkaline phosphatase (41 U/L), g-glutamyl transpeptidase (14 U/L), aspartate aminotransferase (16 U/L), alanine aminotransferase (12 U/L), and bilirubin levels (0.44 mg/dL).

**Figure 1:**
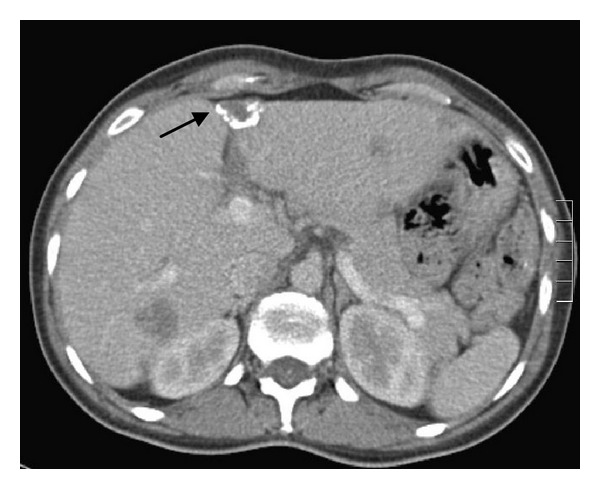
Abdominal CT scan, with intravenous contrast showing several hypodense lesions on both liver lobes. Arrow shows lesion with calcifications.

An abdominal ultrasound was scheduled which showed several hypoechoic solid nodules, regarding both liver lobes, with an irregular echogenic outline and a few of which with multiple calcifications. Immediately afterwards, an abdominal computed tomography was performed which confirmed these lesions and the related calcifications. Specifically, hypodense lesions were seen with the largest of which in the right liver lobe, with a maximum diameter of 3.7 cm (Figure 1). Based on the above, the hypothesis of secondary liver metastasis was proposed.

In the following days, both colonoscopy as well as gastroscopy took place with no pathological findings. Tumor markers including cancer embryonic antigen (CEA), alpha fetoprotein (AFP), and carbohydrate antigen (CA) 19–9 were all within normal limits. Thyroglobulin was measured at 0.2 ng/mL. A follow-up neck ultrasound did not reveal any mass or enlarge lymph nodes, and I^131^ whole body scan did not demonstrate any thyroid remnant nor any metastasis. As part of the patient diagnostic evaluation, in an attempt to find the possible primary tumor, a whole body magnetic resonance imaging (MRI) was performed. The results showed no evidence of an extrahepatic disease.

Percutaneous liver biopsy from the largest liver lesion, guided under computed tomography, showed massive hepatic necrosis. The patient hepatitis serology was negative except from the presence of high anti-HAV IgG antibodies. Furthermore, all blood tests for primary liver disease were also negative.

**Figure 2:**
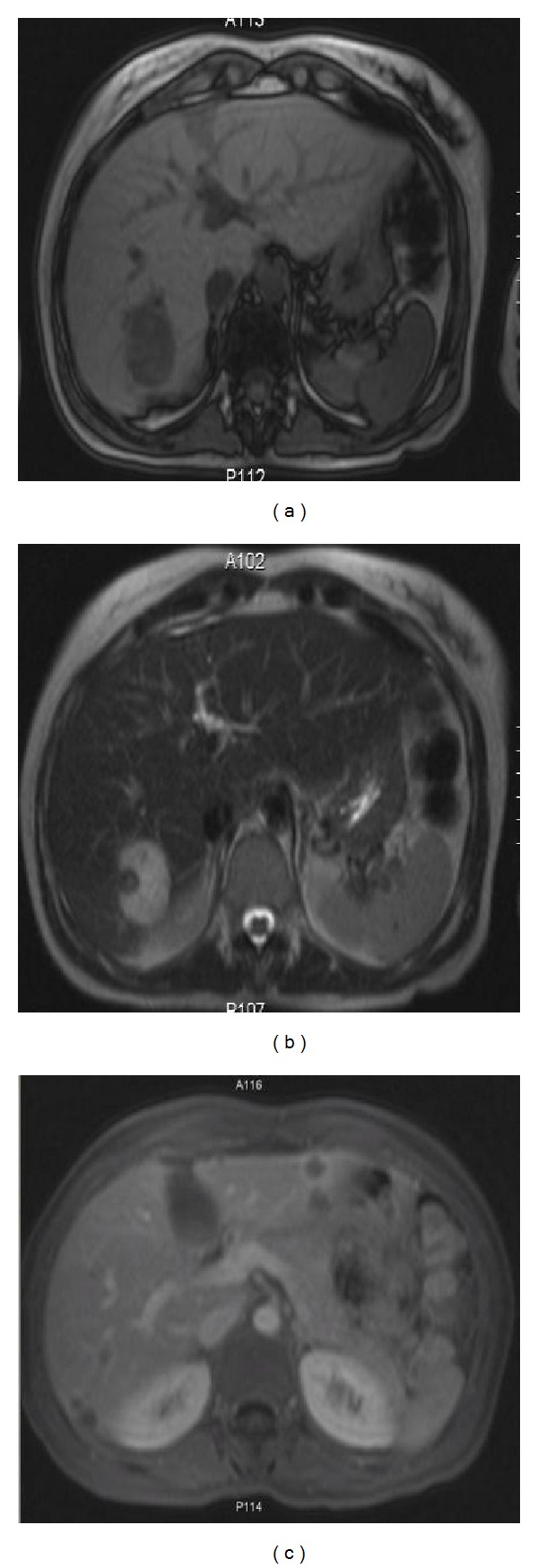
(a) T1-weighted MRI abdomen images showing hypointense lesions. (b) T2-weighted images showing a hyperintense lesion in the right liver lobe. (c) Arrow shows peripheral ring enhancement after intravenous administration of gadolinium.

MRI of the abdomen took place three months after the initial evaluation, which revealed an increase in the maximum diameter of the largest liver lesion, from 3.7 cm to 4.1 cm. The rest of the lesions were without any alterations, in respect to their size. Notably, all lesions in T1-weighted images showed hypointense appearance (Figure 2(a)) in contrast to the T2 hyperintense (Figure 2(b)). After the intravenous administration of gadolinium contrast, all lesions presented a peripheral ring enhancement in T1-weighted images (Figure 2(c)).

Due to the size augmentation of the largest liver lesion, a second liver biopsy was performed, which revealed a well-differentiated hepatocellular carcinoma (HCC).

The presence of normal liver function and normal tumor markers as well as the absence of chronic liver disease or any other HCC predisposing factors drove us to the repetition of the CT-guided liver biopsy for a third time. Once again the biopsy was performed from the largest liver lesion, presented on the right lobe. In contrast to the previous two biopsies, the specimen was analyzed at a more experienced, in liver tumors, histopathology department.

The histopathology report revealed medium- and large-sized pleiomorphic cells that were epithelioid in appearance and that spread within sinusoids and small veins. These cells stained positive for factor VIII-related antigen as well as the endothelial markers CD31 and CD34 (markers for vascular endothelial differentiation). The overall immunohistochemical findings supported the diagnosis of hepatic epitheliod hemangioendothelioma.

Based on the above and the diagnosis achieved, the patient was enlisted and waiting for a liver transplantation.

## 2. Discussion

Epithelioid hemangioendothelioma (EHE) was first described in 1982 by Weiss and Enzinger [1] and since 1982, EHE has been described in many organs, including spleen, bone, brain, meninges, breast, heart, head and neck, soft tissue, stomach, and lymph nodes [1–7]. Hepatic epithelioid hemangioendothelioma (HEHE) was reported first in 1984 by Ishak et al. in a series of 32 patients [8]. The HEHE is a vascular tumor which originates from endothelial cells and is a rare malignancy with an incidence of <0.1 per 100,000 population [9]. This tumor has histologic appearance and behavior between hemangioma and hemangiosarcoma and is classified as a malignant neoplasm by the World Health Organization [10].

HEHE is known to occur in individuals of all ages but is rare in children less than 15 years old. It appears more often in women with a male-to-female ratio, 2 : 3 during the 4th decade of their liver [11].

Until today no definitive etiological factor has been clearly identified for HEHE although several risk factors have been proposed (eg, oral contraceptives, vinyl chloride, asbestos, alcohol, thorotrast, liver trauma, hepatitis virus, alcohol, and chronic liver disease ) [8, 11–17].

Recent studies gave an insight of the genetic basis of EHE. In 2001, Mendlick et al. [18] first revealed an identical chromosomal translocation t(1;3)(p36.3;q25) in two EHE cases. Since then, many studies have confirmed that this specific translocation is unique for EHE among the other epithelioid vascular tumors (epithelioid hemangiomas, epithelioid angiosarcomas) [19–21]. The resulting rearrangement produces a fusion transcript, in which exon 4 of WWTR1 is fused in frame with either exons 8 or 9 of CAMTA1. Both of these genes are known to play important roles in oncogenesis [22–26]. The mechanism by which the fused transcript drives the oncogenesis is unknown, but, probing for translocation t(1;3)(p36.3;q25) through fluorescence in situ hybridization, can serve as a specific diagnostic test of EHE.

The clinical manifestation of HEHE is heterogeneous and varies from asymptomatic to the presence of portal hypertension or hepatic failure [11]. About 25% of the patients have no clinical symptoms when the tumor is first discovered incidentally by imaging studies. Among symptomatic patients, the most common clinical manifestations are nonspecific, including right upper quadrant pain, hepatomegaly, and weight loss [11]. Nonspecific symptoms and the lack of experience of surgeons, radiologists, and histopathologists, due to the rarity of HEHE, make the diagnosis of this entity very challenging. For these reasons approximately 60% to 80% of patients with HEH initially are misdiagnosed [13, 27, 28].

Laboratory parameters are nondiagnostic. 15% of patients do not show any abnormality on blood tests. For the rest 85% of patients, the most common abnormalities are increased alkaline phosphatase (68.6%), g-glutamyl transpeptidase (45.1%), aspartate aminotransferase (28.6%), alanine aminotransferase (23%), and bilirubin (19.9%) [11]. Normal serum alpha-fetoprotein, carcinoembryonic antigen, and cancer antigen 19–9 are typical lab values of patients with HEHE. The only potential role of tumor markers is the excluding of other primary and secondary liver tumors with the limitations of their sensitivity and specificity.

Regarding the imaging studies, the disease can be separated in two subtypes. The nodular subtype is present in early stages and is characterized by the imaging of multifocal nodules. With time, these nodules grow and eventually coalesce, forming large confluent masses preferentially involving the peripheral liver, that is, the diffuse subtype [29].

There exists a great heterogeneity regarding imaging features of HEHE. The liver lesions are typically hypoechoic on US [30, 31], with low density on CT, and are usually hypointense on T1-weighted images and hyperintense on T2-weighted images [11, 32, 33]. Exceptions from the above findings are very frequent. “Capsular retraction sign” (the retraction of the adjacent liver capsule, likely caused by lesion-related fibrosis [29]) in correlation with the “halo” sign after intravenous administration of contrast medium (i.e., the hypointense center and periphery with an intermingled hyperintense layer in between) have been proposed as helpful in an attempt to improve the diagnostic accuracy of this rare hepatic tumor [34].

In our case, imaging features included multiple bilobar hypodense lesions, some of them with calcifications that demonstrated enhancement after the injection of a contrast medium. These findings were in favor with the diagnosis of liver metastases. For this reason we subjected our patient to an extended evaluation including colonoscopy, gastroscopy, evaluation for thyroid cancer liver metastases, because of previous medical history of papillary thyroid cancer, blood test for tumor markers, and magnetic resonance imaging (MRI) of the chest, abdomen, and pelvis, in an effort to find the possible primary tumor.

Definitive diagnosis of epithelioid hemangioendothelioma requires histopathologic examination. Histologically, HEHE appears as nests or cords of epithelioid endothelial cells spreading within sinusoids. Another classical histological feature of these tumors is the presence of intracellular vascular lumina that sometimes contain red blood cells [35]. The diagnosis mostly is confirmed by immunohistochemical evidence of endothelial differentiation. Several well-established endothelial cell markers, such as CD31 (platelet endothelial cell adhesion molecular 1), CD34 (human hematopoietic progenitor cell antigen), and factor VIII-related antigen, are used to confirm epithelioid hemangioendothelioma [13]. Podoplanin, a small mucin-like transmembrane protein that is immunoreactive to D2-40 antibody, is a promising new marker in identifying epithelioid hemangioendothelioma. Although this transmembrane protein is detected in some extrahepatic cancers, it is not expressed in the vascular tumors of the liver except the HEHE [36].

In our case, because of the lack of experience of our histopathology department regarding HEHE, we had missed diagnosis in the first two core biopsies because the possibility of endothelial differentiation was not checked with the appropriate immunohistochemical stains (detection of expression of CD31, CD34, and factor VIII-related antigen). Regarding the first core biopsy, the diagnosis was massive hepatic necrosis, which can be attributed to the central necrosis of the lesion. In the second core biopsy, the results showed evidence of HCC.

The clinical course of epithelioid hemangioendothelioma is highly variable with reports for patients succumb within months after diagnosis in contrast with reports for very milder course such as a report for a patient who was alive 27 years after diagnosis without treatment, and another who had completed spontaneous regression [13, 37].

The need for aggressive treatment of HEHE was documented in a review, which analyzed the survival rates of 434 patients in relation to the given treatment. In this paper, the 5-year survival rates of liver transplantation, local or systemic chemo- and radiotherapy, and no treatment were 55, 30, and 0%, respectively [11].

Although local resection is not excluded from the therapeutic algorithm, it is only feasible in a small portion of patients because the vast majority (81%) of patients have multifocal lesions at the time of diagnosis [11]. The efficiency of liver transplantation as the treatment of choice for HEHE has been documented in two big studies, one from The United States [38] and one from Europe [39, 40]. These studies have showed patient survival rates 5 years after liver transplantation, 64% and 83%, respectively. An unexpected finding was that disease-free survival was not significantly influenced by lymph node status, extrahepatic disease localization, or even vascular invasion.

Other treatment options include chemotherapy, radiotherapy, hormone therapy, thermoablation, and TACE. The experience with these treatments is limited; therefore, the significance of them is difficult to assess.

## 3. Conclusions

Positive imaging findings in addition to certain features, such as occurrence in younger adults, the presence of numerous intrahepatic tumors in patients with a good clinical condition, slow course of the disease, absence of chronic liver disease, normal tumor markers, and normal or mild disturbed laboratory parameters, are suggestive for HEHE. It is mandatory, for these patients, the histopathologist to be experienced in this entity because misdiagnosis, which is not a rare event [11], will drive to a non appropriate treatment with impact to the survival of the patient. In our case if we had adopted the report of the second core biopsy, HCC, the tumor would be classified as unrespectable, the patient would have taken systemic chemotherapy, and she would have lost the opportunity of a liver transplantation and a consequential better survival.
